# Effects of Irbesartan Pretreatment on Pancreatic *β*-Cell Apoptosis in STZ-Induced Acute Prediabetic Mice

**DOI:** 10.1155/2018/8616194

**Published:** 2018-12-02

**Authors:** Cuihong Chen, Li Li, Haijing Qin, Zhenxing Huang, Jing Xian, Jinwei Cai, Yingfen Qin, Jie Zhang, Xinghuan Liang

**Affiliations:** Department of Endocrinology, The First Affiliated Hospital of Guangxi Medical University, Nanning, Guangxi 530021, China

## Abstract

The current study was performed to investigate the effects and potential effects of irbesartan pretreatment on pancreatic *β*-cell apoptosis in a streptozotocin- (STZ-) induced acute mouse model of prediabetes. Twenty-four male BALB/C mice (18–22 g) were randomly divided into three groups: normal control group (NC, *n* = 6), STZ group (STZ, *n* = 8), and irbesartan + STZ group (IRB + STZ, *n* = 10). In the IRB + STZ group, mice were administered irbesartan (300 mg/kg per day) by gavage for one week. The STZ group and IRB + STZ group received STZ (80 mg/kg by intraperitoneal (IP) injection once). The NC group received normal saline (80 mg/kg by IP injection once). Fasting blood glucose prior to STZ injection and presacrifice was analysed using samples withdrawn from the caudal vein to confirm the induction of prediabetes. Haematoxylin and eosin staining, immunohistochemical detection of insulin, and apoptosis analysis were performed. Reverse transcription-quantitative polymerase chain reaction was used to detect angiotensin II type 1 receptor (AT1R), caspase-3, and p38 mitogen-activated protein kinase (MAPK) mRNA expression. Blood glucose was significantly higher in the STZ group (9.01 ± 1.1089 vs 4.78 ± 0.7026) and IRB + STZ group (7.86 ± 1.1811 vs 4.78 ± 0.7026) compared with the NC group (*P* < 0.05). In comparison to the STZ group, the islet cell damage was marginally improved in the IRB + STZ group, and the IRB + STZ group had a significantly lower apoptotic rate than the STZ group (22.42 ± 8.3675 vs 50.86 ± 5.3395, *P* < 0.001). AT1R expression in the IRB + STZ group was lower than that in the STZ group (1.56 ± 1.2207 vs 3.92 ± 2.4392, *P* < 0.05). The mRNA expression of caspase-3 in pancreatic tissue was significantly lower in the IRB + STZ group than in the STZ group (0.90 ± 0.7272 vs 1.88 ± 1.0572, *P* < 0.05). Similarly, the IRB + STZ group also had lower p38MAPK levels than the STZ group (1.16 ± 1.0642 vs 2.55 ± 1.7925, *P* > 0.05). In conclusion, irbesartan pretreatment improved glucose levels and insulin secretion and decreased islet *β*-cell apoptosis to protect islet *β* cells in an STZ-induced acute prediabetic mouse model.

## 1. Introduction

Previous clinical studies have indicated that blockade of the renin-angiotensin system (RAS) using inhibitors of angiotensin-converting enzymes (ACEIs) or angiotensin-receptor blockers (ARBs) reduces the occurrence of diabetes and the incidence of complications associated with diabetes in hypertensive patients [1–4]. Similarly, patients with hypertension treated with irbesartan not only reduced blood pressure, but also improved metabolic parameters, for example, blood glucose and lipid levels as well as liver function [5]. These studies suggest that ACEIs or ARBs may be beneficial for the prevention of diabetes. However, the underlying mechanisms involved remain unclear.

Irbesartan, a commonly used ARB, has been reported to have various beneficial effects beyond reduction in blood pressure. Clinical and experimental data indicate that irbesartan can ameliorate diabetic nephropathy by suppressing signalling from the receptor activator of nuclear factor kappa B (NF-*κ*B) [6]. Additionally, irbesartan may have a role in protecting against diabetes-related bone damage by blocking the detrimental effects of advanced glycation end product- (AGE-)/receptor for advanced glycation end product- (RAGE-) mediated oxidative stress [7]. Furthermore, irbesartan treatment can improve diabetes-related arteriosclerosis [8]. In the diabetic Zuker rat model with insulin-resistant obesity induced by fructose, irbesartan treatment led to improved glucose tolerance and insulin sensitivity [9]. Thus, irbesartan is a widely used ARB in clinical practice, especially in hypertensive patients with diabetes, and it is important to explore the effect of irbesartan in the prevention of diabetes further.

Prediabetes is the stage before DM in which not all of the symptoms or signs required to diagnose diabetes are present, but blood glucose is abnormally high. Epidemiological data shows that the estimated prevalence of prediabetes in China was much higher in 2013 than the estimate in 2008 (35.7% vs 15.5%) [10]. Prediabetes is often associated with obesity, hypertension, and dyslipidaemia. There are many studies that have investigated the effects of ACEI or ARB in diabetic models; however, as the treatment for diabetes is limited to the later stages when a diagnosis has been received, and even patients with well-controlled blood glucose gradually develop diabetes-related complications, it is vital to identify preventative methods. Hypertensive patients often have impaired glucose tolerance [11], and ACEIs and ARBs have both been reported to help prevent diabetes in patients with hypertension. Thus, the preventative function of ACEI or ARB in prediabetes should also be explored, as few studies have investigated their effects in this context.

The RAS is a vital determinant of blood pressure and intravascular volume. The major components involved in the system are renin, angiotensinogen, angiotensin-converting enzyme (ACE), angiotensin-converting enzyme 2, angiotensin II (Ang II), and its receptors Ang II type 1 receptor (AT1R) and Ang II type 2 receptor (AT2R) [12]. In addition to the classical (circulating) RAS, there is a growing body of experimental evidence indicating that local RAS (complete or partial) exists in several tissues and has pivotal roles in a variety of developmental and disease processes [13]. Evidence has suggested that the pancreas has a local RAS in humans, rats, mice, and dogs, with expression of the essential RAS components, including Ang II and its receptors (AT1R and AT2R), detected in the pancreas. In the human pancreas, expression of AT1 receptors is confined to the *β* cells and endothelial cells of the islets. Emerging evidence suggests that the local RAS in pancreatic islets has functions in glucose homeostasis. In rats, islet blood flow appears to be suppressed by locally produced Ang II under normal conditions. In vitro, Ang II delays the first phase of insulin release in response to glucose. These findings suggest that locally generated Ang II may indirectly affect glucose homeostasis via somatostatin-mediated inhibition of insulin or glucagon secretion under certain conditions [12, 14, 15]. Ang II is a key bioactive peptide of the RAS. Initially, renin cleaves its substrate, angiotensinogen, to form the decapeptide angiotensin I (Ang I). Then, the two terminal amino acids are split by the biologically active dipeptide carboxypeptidase ACE to form the octapeptide Ang II. Ang II exerts its biological actions via two G-protein-coupled receptors, AT1R and AT2R, with the deleterious effects predominantly mediated via AT1R [16].

Emerging data support that chronic hyperglycaemia, AGEs, high fat, obesity, inflammation, and hypertension can activate the pancreatic RAS by increasing AT1R expression, inflammation, oxidative stress, and apoptosis in pancreatic *β* cells [17, 18]. Previous studies have illustrated the novel roles of the pancreatic RAS in pancreatic *β*-cell function. In diabetes, activation of the pancreatic RAS by hyperglycaemia and hyperlipidaemia can result in reduced insulin biosynthesis, insulin secretion, and islet blood flow, and also increased *β*-cell apoptosis, oxidative stress, and islet fibrosis [14, 19]. In our previous study, we demonstrated that high glucose promotes islet cells apoptosis in vitro [20]. However, the underlying mechanisms are poorly elucidated. High glucose activates the RAS and upregulates Ang II and AT1R expression. Ang II induces the production and activation of reactive oxygen species (ROS) via AT1R-mediated nicotinamide-adenine dinucleotide phosphate (NADPH) oxidase and increases the expression of the apoptosis-related factors Bax and caspase-3 [20].

Understanding the relationship between the RAS and pancreatic *β*-cell function during prediabetes and identifying individuals that are at risk of developing specific pathologies will guide more effective personalized prevention and treatment. Therefore, the present study was aimed at investigating the effect of irbesartan pretreatment on pancreatic *β*-cell apoptosis in a streptozotocin- (STZ-) induced acute prediabetic mice.

## 2. Materials and Methods

### 2.1. Animals

Male BABL/C mice weighing approximately 18–22 g were purchased from the Animal Center of Guangxi Medical University (Nanning, China). Mice were humanely housed at 22 ± 2°C with 12-h light/dark cycles. All animals had free access to food and water. All animal studies were approved by the ethical review committee of Guangxi Medical University and followed the regulations of the National Institutes of Health guidelines on the care and welfare of laboratory animals.

### 2.2. Drugs and Chemicals

Irbesartan was obtained from Sanofi (S.A., Paris, France), and STZ was purchased from Sigma-Aldrich Inc., (St. Louis, MO, USA). The appropriate pretreated dose of irbesartan was determined in preliminary experiments (supplemental materials, part 1).

### 2.3. Induction of Experimental Prediabetes

An acute prediabetic mouse model was successfully established using STZ in a previous study [21]. Prediabetes was induced in mice by a single intraperitoneal injection of STZ (80 mg/kg). Mice with mild hyperglycaemia (fasting glucose before sacrifice > 6 mmol/L and <16.7 mmol/L), mild pancreatic islet damage, and <60% apoptosis of pancreatic *β* cells were considered to indicate prediabetes, and these mice were included in the study.

### 2.4. Study Design

The animals were randomly divided into three groups: normal control group (NC group, *n* = 6), STZ group (STZ, *n* = 8), and irbesartan + STZ group (IRB + STZ, *n* = 10). In the IRB + STZ group, mice were administered irbesartan (300 mg/kg per day) orally by gavage for one week. The STZ group and IRB + STZ group received STZ (80 mg/kg by intraperitoneal (IP) injection once); the NC group received normal saline (80 mg/kg by IP injection once). Fasting blood glucose was taken before STZ injection and before sacrifice using blood from the caudal vein, and was used to confirm the induction of prediabetes. Mice were sacrificed by cervical dislocation after 12 h.

### 2.5. Haematoxylin and Eosin (H&E) Staining

Isolated pancreas samples were fixed in 4% formaldehyde. The tissues were sectioned and stained with H&E for morphological analysis.

### 2.6. Immunohistochemical Detection

Before dewaxing, the tissue sections were placed in a 60°C incubator. Following dewaxing and hydration, the tissue sections were immersed into xylene and then transferred to ethanol. The sections were incubated in 0.01 mol/L citrate buffer (pH 6.0) for antigen retrieval. Then, freshly prepared 3% hydrogen peroxide solution was added to eliminate endogenous peroxidase activity. Next, the sections were incubated with anti-insulin antibody (cat. no. BM0080; Wuhan Boster Biological Technology Ltd., Wuhan, China; 1 : 200), then incubated with a secondary antibody, and finally counterstained with haematoxylin.

### 2.7. Terminal Deoxynucleotidyl Transferase dUTP Nick End Labelling (TUNEL) Assay

Tissues were sectioned as described above. The assay was performed according to the instructions of the TUNEL kit (cat. no. 11684817910; Roche Diagnostics, Basel, Switzerland). The sections were washed in PBS three times before incubation in diaminobenzidine for 3 min and analysed in mounting medium under a fluorescence microscope. The number of positive cells in five noncontinuous high power fields of vision was observed and counted under the microscope.

### 2.8. Reverse Transcription-Quantitative Polymerase Chain Reaction (RT-qPCR)

Total RNA was isolated from pancreas samples using TRIzol, and RT was then performed using the PrimeScript®RT reagent kit (cat. no. HRR037A; Takara Biotechnology Co. Ltd., Dalian, China). qPCR analysis was performed using a Mastercycler PCR machine (Mastercycler® X50s; Eppendorf, Hamburg, Germany). The relative quantification analysis for a given gene was performed using 7500 Fast Real-Time PCR (Applied Biosystems; Thermo Fisher Scientific Inc., Waltham, MA, USA). Oligonucleotide sequences are provided in the supplemental materials, part 2.

### 2.9. Statistical Analyses

SPSS 18.0 statistical software (SPSS Inc., Chicago, IL, USA) was used for statistical analysis. Continuous data with a normal distribution is expressed as the mean ± standard deviation (*x* ± *s*). Analysis of variance was used to compare the means of multiple samples. When the data met the assumption of homogeneity of variances, the least significant difference test was used for the comparison of multiple samples. When the data did not meet the homogeneity of variance assumption, Games-Howell was used to compare multiple samples. *P* < 0.05 was considered to indicate statistical significance.

## 3. Results

### 3.1. Changes in Blood Glucose

**Figure 1:**
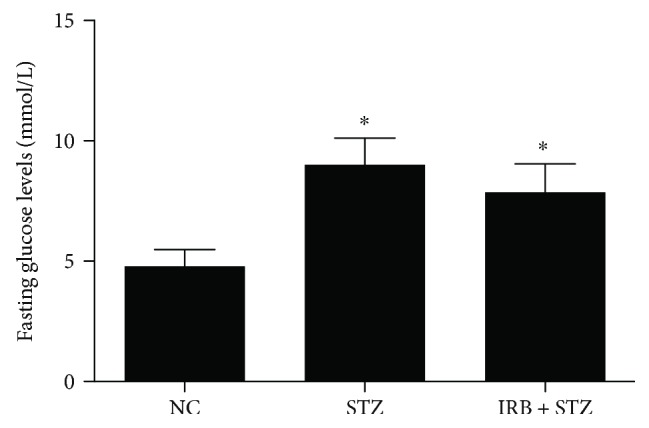
Fasting glucose levels after 12-hour intervention in each group. NC = normal control group (*n* = 6, received normal saline 80 mg/kg by intraperitoneal (IP) injection once), STZ = streptozotocin group (*n* = 8, received STZ 80 mg/kg by IP once), and IRB + STZ = irbesartan + streptozotocin group (*n* = 10, administered irbesartan 300 mg/kg per day by gavage for one week before receiving STZ 80 mg/kg by IP once). Data are presented as mean ± SD. ∗ compared with the NC group, *P* < 0.05.

Compared with the control group, blood glucose was significantly increased in the STZ group (9.01 ± 1.1089 vs 4.78 ± 0.7026) and IRB + STZ group (7.86 ± 1.1811 vs 4.78 ± 0.7026) after injection of STZ (*P* < 0.001); however, all blood glucose levels were <10 mmol/L. The level of blood glucose elevation in the IRB + STZ group was smaller than that in the STZ group, but there was no significant difference between the two groups (Figure 1 and Table 1).

### 3.2. Effect of Irbesartan on Islet Morphology

**Figure 2:**
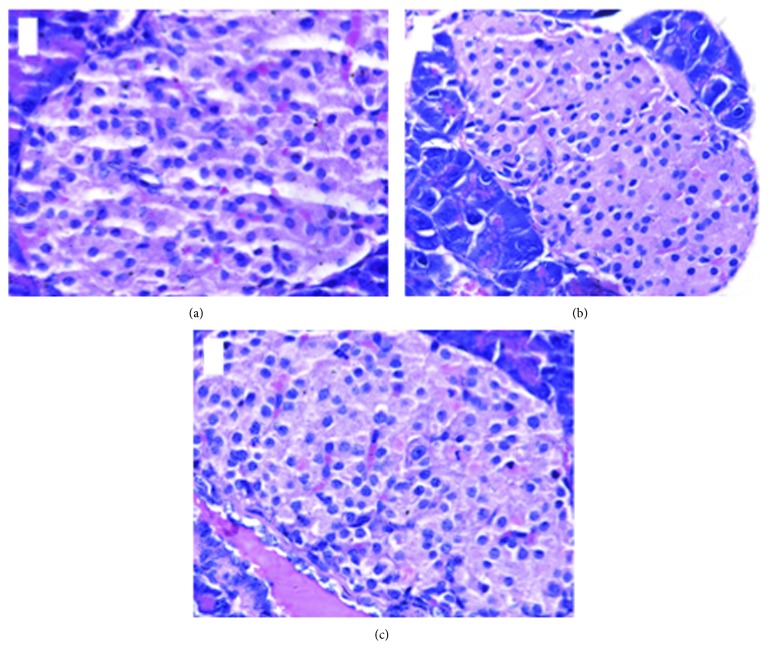
The HE staining of islet cells in mice in each group (400x). (a) NC group; (b) STZ group; and (c) IRB + STZ group.

In the NC group, the islet cells were clumped together with abundant capillaries between the groups of cells. The morphology of islet cells was normal, and the nuclei were round and clearly visible. After STZ injection, islet cells appeared swollen with hyaline degeneration, nuclear dissolution, and nuclear condensation. In comparison to the STZ group, the islet cells damage were marginally improved in the IRB + STZ group (Figure 2).

### 3.3. Apoptosis Is Reduced by Irbesartan in the Pancreas of STZ-Induced Mice

**Figure 3:**
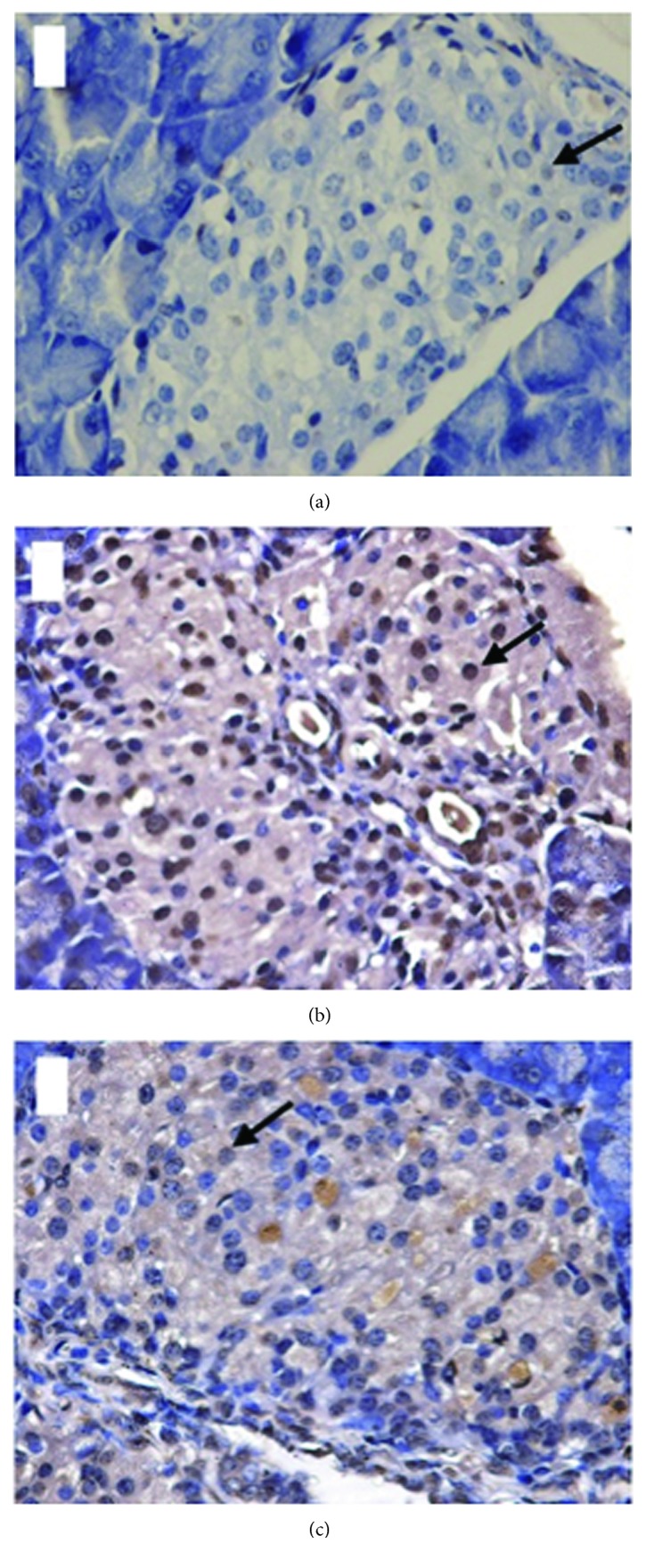
Apoptosis of islet cells in mice in each group (400x). The arrow pointed to the apoptosis islet cells. (a) NC group; (b) STZ group; and (c) IRB + STZ group.

**Figure 4:**
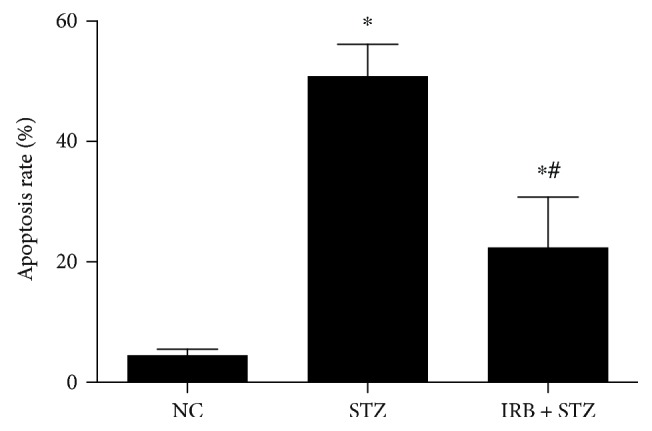
Apoptosis rate of pancreatic islet cells in each group. NC = normal control group (*n* = 6, received normal saline 80 mg/kg by intraperitoneal (IP) injection once), STZ = streptozotocin group (*n* = 8, received STZ 80 mg/kg by IP once), and IRB + STZ = irbesartan + streptozotocin group (*n* = 10, administered irbesartan 300 mg/kg per day by gavage for one week before receiving STZ 80 mg/kg by IP once). Data are presented as mean ± SD. ∗ compared with the NC group, *P* < 0.05. # compared with the STZ group, *P* < 0.05.

Compared with the NC group, the apoptotic rates were significantly higher (*P* < 0.001) in the STZ group (50.86 ± 5.3395 vs 4.53 ± 1.0020) and IRB + STZ group (22.42 ± 8.3675 vs 4.53 ± 1.0020) were significantly higher (*P* < 0.001). However, the IRB + STZ group had a significantly lower rate of apoptosis compared with the STZ group (22.42 ± 8.3675 vs 50.86 ± 5.3395, *P* < 0.001), indicating that irbesartan preconditioning can partially protect islet cells from STZ by reducing the apoptotic rate of islet cells (Table 2, Figures 3 and 4).

### 3.4. Irbesartan Increases Insulin Levels in STZ-Induced Mice

**Figure 5:**
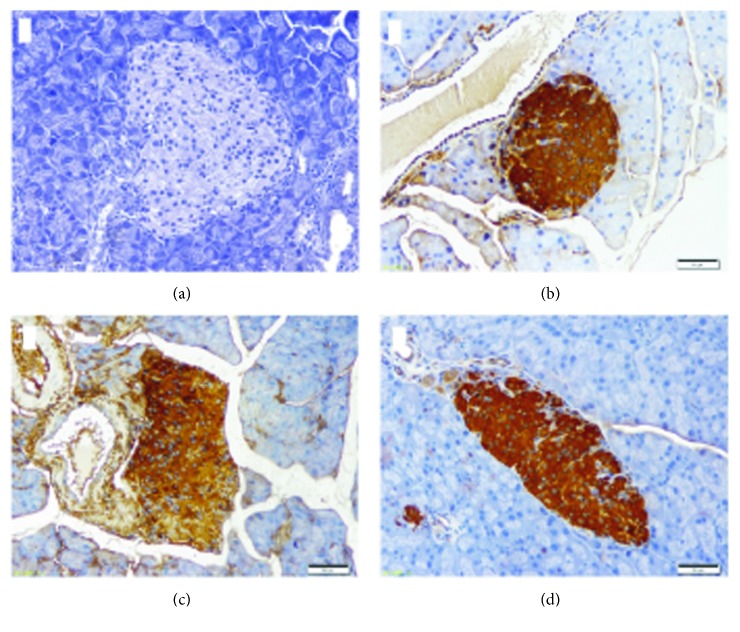
Immunohistochemical staining of insulin in pancreas of mice in each group (200×). (a) Negative control group; (b) normal control group; (c) STZ group; and (d) IRB + STZ group.

The level of insulin in the islet *β*-cells was significantly decreased in the STZ group compared with the NC group (0.44 ± 0.0423 vs 0.34 ± 0.0410, *P* < 0.001), indicating that STZ induced damage of pancreatic *β* cells, resulting in reduced insulin reserves. In the IRB + STZ group, insulin expression was higher than in the STZ group (0.38 ± 0.0315 vs 0.34 ± 0.0410, *P* < 0.001), but still lower than in the NC group (0.38 ± 0.0315 vs 0.44 ± 0.0423, *P* < 0.001) (Table 3 and Figure 5).

### 3.5. Effect of Irbesartan on Gene Expression

**Figure 6:**
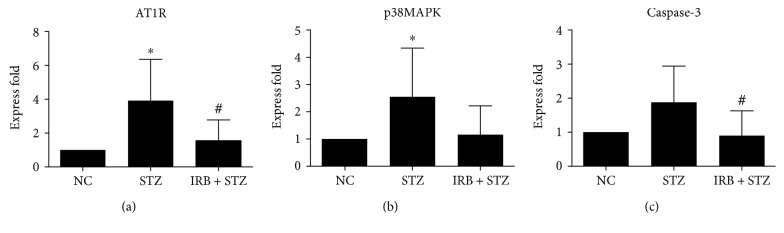
AT1R (a), p38MAPK (b), and caspase-3 (c) mRNA expression levels in pancreas in each group. NC = normal control group (*n* = 6, received normal saline 80 mg/kg by intraperitoneal (IP) injection once), STZ = streptozotocin group (*n* = 8, received STZ 80 mg/kg by IP once), and IRB + STZ = irbesartan + streptozotocin group (*n* = 10, administered irbesartan 300 mg/kg per day by gavage for one week before receiving STZ 80 mg/kg by IP once). Data are presented as mean ± SD. ∗ compared with the NC group, *P* < 0.05. # compared with STZ group, *P* < 0.05.

The AT1R mRNA expression was significantly increased in pancreatic tissue of the STZ group compared with the NC group (*P* < 0.05), suggesting that the pancreatic RAS was activated during apoptosis of pancreatic islet cells. Furthermore, ATIR expression was lower in the IRB + STZ group compared with the STZ group (1.56 ± 1.2207 vs 3.92 ± 2.4392, *P* < 0.05). There was approximately double the caspase-3 mRNA expression in the STZ group compared with the NC group (*P* > 0.05), suggesting that apoptosis of islet cells was induced by STZ. Furthermore, the mRNA expression of caspase-3 in pancreatic tissue was significantly lower in the IRB + STZ group than in the STZ group (0.90 ± 0.7272 vs 1.88 ± 1.0572, *P* < 0.05). Similarly, the IRB + STZ group also exhibited reduced p38 mitogen-activated protein kinase (MAPK) levels compared with the STZ group (1.16 ± 1.0642 vs 2.55 ± 1.7925, *P* > 0.05), although there was not up to statistical difference, further indicating that irbesartan attenuated apoptosis of pancreatic *β* cells (Table 4 and Figure 6).

## 4. Discussion

The most intriguing findings of the present study include that irbesartan pretreatment exhibited the following benefits in the prediabetic model: (1) improved blood glucose levels; (2) minimized the damage to pancreatic cells; (3) reduced pancreatic *β*-cell apoptosis; (4) increased insulin levels in the pancreatic *β* cells; and (5) reduced the expression of AT1R, p38MAPK, and caspase-3.

A previous clinical study suggested that when patients are diagnosed with diabetes the number of pancreatic *β* cells has been reduced by 50%, with *β*-cell apoptosis as the main cause of the loss of *β* cells [22], which also indicates that *β*-cell apoptosis is the main pathological change in the prediabetic stage. If appropriate interventions are used at this stage, the prevalence of diabetes may significantly decrease and high-risk populations will benefit from preventative treatment. However, few studies have investigated the physiological and pathological processes of prediabetes. Thus, we first established a mouse model of prediabetes in this study. Our previous study demonstrated that a small dose of STZ can acutely induce *β*-cell apoptosis as a nondiabetic mouse model, which we consider to be a prediabetic model for the following reasons: (1) mild hyperglycaemia; (2) mild pancreatic islet damage; and (3) apoptosis of pancreatic *β* cells at <60% [21]. Thus, we were able to investigate the role of irbesartan in the prevention of diabetes.

A large body of data indicates that a local RAS exists in the pancreatic islet cells in both humans and several other animals. Activation of the pancreatic RAS, mainly through AT1R, acutely diminishes insulin secretion, which is due to reduced proinsulin biosynthesis and islet blood flow. The RAS also causes islet apoptosis and fibrosis, and may consequently contribute to islet cell dysfunction in type 2 diabetes [23]. It is well documented that RAS blockade using ACEIs or ARBs has a positive effect on the development processes of diabetes. For instance, treatment with an ACEI in diabetic mice reduced circulating glucose [18]. However, no studies have investigated the effects of RAS blockade in prediabetes. To the best of our knowledge, this is the first study to explore the influence of ARB irbesartan pretreatment on pancreatic *β*-cell function. Consistent with previous studies, the findings of the present study suggested that treatment of irbesartan prior to STZ intervention reduced the STZ-induced increase in blood glucose in the prediabetic mouse model, which provided experimental evidence indicating that irbesartan can improve glucose levels, not only in diabetes, but also in prediabetes.

The mechanisms by which ACEIs or ARBs improve glucose status and pancreatic *β*-cell function have not been fully elucidated. RAS blockade is associated with increased *β*-cell mass, improvement in first-phase insulin secretion, and normalization of islet morphology [24]. Furthermore, in a previous study, islet fibrosis was diminished and *β*-cell dysfunction was improved following treatment of rats with the ACE inhibitor ramipril for 24 weeks [17]. Similarly, other evidence from animal models has demonstrated that ACEIs and ARBs prevent the development of fibrosis [25]. Furthermore, ARBs and ACEIs increase glucose transporter (GLUT) 4 translocation to the membrane, resulting in increased skeletal muscle glucose uptake and insulin sensitivity in animal models [18, 26]. The present study provided further evidence that pretreatment with irbesartan attenuates pancreatic damage, reduces islet *β*-cell apoptosis, and increases insulin secretion, which affirms that irbesartan can prevent STZ-induced pancreatic islet damage in an acute prediabetic mouse model.

Emerging data support the notion that elevated glucose level and other factors can stimulate the activity of RAS components, such as Ang II and AT1R. Isolated human islets exposed to high glucose concentrations exhibited enhanced expression of AT1R and ACE [27]. Notably, Ang II is involved in lipid- and glucose-induced inflammation, oxidative stress, and apoptosis via AT1R [18]. Pancreatic *β* cells have relatively low levels of antioxidants; thus, increased oxidative stress is more likely to induce apoptosis and decrease *β*-cell numbers [1]. There is a vicious circle, in which activation of the RAS, amyloid deposition, and oxidative stress-related changes result in elevated apoptosis, reduction of *β*-cell mass, and impairment of insulin secretion and insulin production, which in turn impairs lipid and carbohydrate homeostasis. Finally, lipotoxicity and glucotoxicity further deteriorate *β*-cell dysfunction and insulin resistance [27]. In an STZ-induced pancreatic *β*-cell apoptosis model, STZ increases the synthesis of ROS, causes mitochondrial damage, and reduces the formation of adenosine triphosphate in the pancreas, thus, resulting in islet *β*-cell damage [28]. The transfer of the methyl group from STZ to DNA causes damage, which induces a chain of events that results in the fragmentation of the DNA [28]. Our present study was aimed at exploring the effects of irbesartan in preventing diabetes, and to determine whether the changes discussed were also present in prediabetes. Therefore, irbesartan pretreatment was performed prior to induction of an STZ-induced prediabetes model in mice to imitate the preventative use of ACEI or ARB in a clinical setting. Following injection of STZ, the expression of AT1R in the STZ group was markedly increased compared with that in the NC group, but this effect was attenuated by irbesartan pretreatment, indicating that the local pancreatic RAS was active in the prediabetic model, and irbesartan can diminish the activation of local pancreatic RAS.

Research has focused on the influence of ACEIs or ARBs on the pancreas and pancreatic *β*-cell function; however, the mechanistic pathway that mediates the effects is still poorly understood. Currently, the evidence indicates that two pathways may be involved: MAPK and caspase pathways. The signalling events in response to Ang II activate redox-sensitive signal molecules, such as MAPKs [29]. MAPKs are a family of serine/threonine kinases associated with the pathogenesis of vascular fibrosis and hypertension. In addition, MAPKs are also crucial regulatory proteins that control the cellular response to apoptosis, growth, and stress signals [30]. Key MAPKs include p38MAPK, extracellular signal-regulated kinases 1/2, and stress-activated protein kinase/c-Jun N-terminal kinases [31]. Particularly, p38MAPK is a vital component of the oxidative stress-sensitive pathways activated by Ang II in vascular smooth muscle cells, and it is also a key protein crucial for various cellular processes, including cell differentiation, inflammation, cell growth, and cell death [32, 33]. Furthermore, Ang II can induce ROS generation via oxidative stress reactions, which in turn, activates the p38MAPK signalling pathway. Both p38MAPK and ROS molecules are involved in myocardial fibrosis formation [34]. Caspases are a unique class of aspartate-specific proteases that are the key components of the apoptotic response. Caspase-3 has a pivotal role in the intrinsic and extrinsic apoptotic pathways [35], and Ang II has been reported to stimulate caspase-3 activity [36]. The present study found that the early intervention with irbesartan reduced the STZ-induced activation of apoptosis signal pathways (p38MAPK and caspase-3) in prediabetes. We speculate that irbesartan blocked the effects of STZ-induced prediabetes by inhibiting Ang II and AT1R, and thus reducing the activation of the p38MAPK and caspase-3 pathways, ultimately improving pancreatic *β*-cell function in the acute prediabetic stage.

## 5. Conclusions

In summary, irbesartan pretreatment improved glucose levels and insulin secretion in the pancreatic islet and decreased islet *β*-cell apoptosis to protect islet *β* cells in an STZ-induced acute prediabetic mouse model. The underlying mechanism may be that irbesartan blocks activation of the pancreatic RAS by inhibiting AT1R expression, leading to decreased activity of p38MAPK and caspase-3-mediated apoptosis pathways. These actions protect the pancreatic islet *β* cells and may have a diabetes prevention effect. However, this study only tentatively explores the molecular changes that are associated with the effects of irbesartan during prediabetes. The specific molecular mechanisms and signal pathways still require further investigation.

## Figures and Tables

**Table 1:** Fasting glucose levels after 12-hour intervention in each group.

Group	*n*	Glucose level (mmol/L)
NC	6	4.78 ± 0.7026
STZ	8	9.01 ± 1.1089^∗^
IRB + STZ	10	7.86 ± 1.1811^∗^

Notes: data are presented as mean ± SD. ∗ compared with the NC group, *P* < 0.05.

**Table 2:** Apoptosis rate of pancreatic islet cells in each group.

Group	*n*	Apoptosis (%)
NC	6	4.53 ± 1.0020
STZ	8	50.86 ± 5.3395^∗^
IRB + STZ	10	22.42 ± 8.3675^∗^^**#**^

Notes: data are presented as mean ± SD. ∗ compared with the NC group, *P* < 0.05. # compared with the STZ group, *P* < 0.05.

**Table 3:** Insulin expression in the pancreas of mice in each group.

Group	*n*	Insulin
NC	6	0.44 ± 0.0423
STZ	8	0.34 ± 0.0410^∗^
IRB + STZ	10	0.38 ± 0.0315^∗^^#^

Notes: data are presented as mean ± SD. ∗ compared with the NC group, *P* < 0.05. # compared with the STZ group, *P* < 0.05.

**Table 4:** AT1R, caspase-3, and p38MAPK expression levels in pancreas in each group.

Group	*n*	AT1R	Caspase-3	p38MAPK
NC	6	1	1	1
STZ	8	3.92 ± 2.4392^∗^	1.88 ± 1.0572	2.55 ± 1.7925^∗^
IRB + STZ	10	1.56 ± 1.2207^#^	0.90 ± 0.7272^#^	1.16 ± 1.0642

Notes: data are presented as mean ± SD. ∗ compared with the NC group, *P* < 0.05. # compared with the STZ group, *P* < 0.05.

## Data Availability

The data used to support the findings of this study are included within the article and the supplementary information file.

## Supplementary Materials

Supplementary Materials Preliminary experiments of the appropriate pretreated dose of irbesartan are provided in supplemental materials, part 1. Oligonucleotide sequences are provided in supplemental materials, part 2.
