# Supplementary material for: Effects of Irbesartan Pretreatment on Pancreatic β-Cell Apoptosis in STZ-Induced Acute Prediabetic Mice
Source: Oxid Med Cell Longev. 2018 Dec 2;2018:8616194. doi: 10.1155/2018/8616194 (PMC6304884; doi:10.1155/2018/8616194)
Supplement: Supplementary Materials — Preliminary experiments of the appropriate pretreated dose of irbesartan are provided in supplemental materials, part 1. Oligonucleotide sequences are provided in supplemental materials, part 2. [file 8616194.f1.docx]

**Supplemental materials**

Part 1. Exploration of the duration and dose of irbesartan pretreatment in a mouse model.

As far as we know, there is no study using irbesartan as a pretreated drug in an animal model. Most studies used it as a treated drug. Thus, we had to explore the duration and dose of irbesartan pretreatment in a mouse model.

We firstly discussed the duration of irbesartan pretreatment. The experiment was divided into two groups, one-week group and two-week group，both groups had the same dose of irbesartan. At the end of each pretreatment, the two groups were given intraperitoneal injection of STZ 80 mg/kg. After STZ injection of 12h, the mice are weighed, blood samples were collected for blood glucose detection. The results showed that there was no significant difference in mice weight between these two groups. However, there was a statistically significant difference in blood glucose between the two groups, the glucose levels in one-week group was markedly higher than two-week group (Supplemental table1, figure 1), which indicated that the longer the irbesartan pretreatment, the stronger the hypoglycemic effect of irbesartan accumulated in mice in STZ-induced hyperglycemia. If we chose two weeks as the duration of irbesartan pretreatment, we might fail to established a prediabetic mouse model eventually since the accumulated irbesartan in mice were too much to keep a mild hyperglycemia status or there was just mild elevation of blood glucose, but no changes in pancreatic islet histology. Hence, in our formal study of this manuscript, mice were given irbesartan pretreatment for one week.

Then, we explored the dose of irbesartan pretreatment. In the literatures on irbesartan as a therapeutic drug, most of them selected a doses of irbesartan 50mg/kg , and long-term intervention (one to several months)。Irbesartan was used as a pretreated drug in our study, and our model was an acute model, the dose of irbesartan should be different from previous studies’. According to the instruction of irbesartan, the recommended dose is 150mg to 300mg per day. Therefore, the experiment was divided into five groups. Irbesartan was given at a dose of 100mg/kg, 200mg/kg, 300mg/kg,400mg/kg,500 mg/kg respectively. After one-week intervention, each group was given intraperitoneal injection of STZ 80 mg/kg. After STZ injection of 12h, the mice are weighed, blood samples were collected for blood glucose detection. The results showed that there was no significant difference in body weight and blood sugar among the five groups (Supplemental table 2). When selected dose, we tried to select the dose that increased blood glucose significantly to ensure the success of modeling later. In this process, we abandoned the highest glycemic group, the 100mg/kg group, because there might be possible that blood glucose levels in mice of this group were elevated significantly to reach a standard of diabetes, and failed to establish a model of prediabetes eventually. Finally, we chose 300mg/kg as a dose of irbesartan pretreatment in our formal experiment.

To sum up, we eventually chose irbesartan 300mg/kg pretreatment for one week in our formal experiment.

Supplemental table 1. Blood glucose levels and body weight at different pretreated time of STZ

| intervention time |  | N | glucose(mmol/L) | weight(g) |
| --- | --- | --- | --- | --- |
| 1 week |  | 10 | 8.97±0.78 | 21.82±1.63 |
| 2week |  | 10 | 7.55±0.62 | 21.74±0.91 |
|  |  |  |  |  |
| t |  |  | 4.514 | 0.135 |
| p value |  |  | 0.000 | 0.894 |


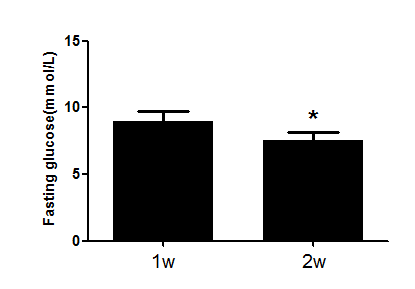


Supplemental figure 1. Blood glucose levels at different pretreated time of STZ

* compared with one-week group，blood glucose reduced significantly in two-week group, p<0.001

Supplemental table 2. Blood glucose levels and body weight at different pretreated dose of irbesartan at one week

| irbesartan dose(mg/kg) |  | N | glucose(mmol/L) | weight(g) |
| --- | --- | --- | --- | --- |
| 100 |  | 2 | 9.50±1.13 | 21.10±0.28 |
| 200 |  | 2 | 8.40±0.00 | 20.20±0.85 |
| 300 |  | 2 | 9.20±0.99 | 21.90±1.41 |
| 400 |  | 2 | 8.65±0.64 | 22.40±2.55 |
| 500 |  | 2 | 9.10±1.13 | 23.50±1.41 |
|  |  |  |  |  |
| F |  |  | 0.493 | 1.398 |
| p value |  |  | 0.744 | 0.355 |

Part 2. Primer sequences for real-time polymerase chain reaction (PCR)

Supplemental table 3 Primer sequences for real-time polymerase chain reaction (PCR)

| Target mRNA Primer | Primer sequence | Product (bp) |
| --- | --- | --- |
| AT1R | 5’ 5'-TGGGCGTCATCCATGACTGTA -3' | 182 |
|  | 3’ 5'-TGAGTGCGACTTGGCCTTTG -3' |  |
| Caspase-3 | 5’ 5'-AGGCAAGGTGCTAAAATCCATC -3' | 233 |
|  | 3’ 5'-TCAAACCACATTCTCTCCAACTACA -3' |  |
| P38 MAPK | 5’ 5'-CTGTCGAGACCGTTTCAGTCCA -3' | 112 |
|  | 3’ 5'-GTGTGAACACATCCAACAGACCAA -3' |  |
| GAPDH | 5’ 5'-TGTGTCCGTCGTGGATCTGA -3' | 150 |
|  | 3’ 5'-TTGCTGTTGAAGTCGCAGGAG -3' |  |
